# Seed Proteomic Profiles of Three *Paeonia* Varieties and Evaluation of Peony Seed Protein as a Food Product

**DOI:** 10.1155/2020/5271296

**Published:** 2020-11-18

**Authors:** Xiuxia Ren, Yantong Shi, Yuqian Xue, Jingqi Xue, Yuanyuan Tian, Shunli Wang, Xiuxin Zhang

**Affiliations:** ^1^Key Laboratory of Biology and Genetic Improvement of Horticultural Crops, Ministry of Agriculture and Rural Affairs, Beijing, China; ^2^Institute of Vegetables and Flowers, Chinese Academy of Agricultural Sciences, Institute of Peony, Chinese Academy of Agricultural Sciences, Beijing 100081, China; ^3^Beijing Agricultural Technology Extension Station, Beijing 100029, China; ^4^National Agricultural Science & Technology Center, Chengdu, China

## Abstract

Peony (*Paeonia*) has high ornamental, edible, and medicinal values. In order to distinguish seeds varieties, describe the proteomic profiles correlated with stress tolerance, and evaluate peony seed protein (PSP) as a functional food product, we characterized the seed protein profiles of these three species and their glucosidase inhibition activities. Results showed that the intensity of protein bands in sodium dodecyl sulfate polyacrylamide gel electrophoresis (SDS-PAGE) and specific protein ID (especially for specifically expressed proteins (SEPs)) was effective to distinguish these peony seed varieties. Proteomic analysis of the three species showed that *P. ostii* “Fengdan” has heat and pathogen tolerance-related proteins, while *P. rockii* has higher content of proteins related to cold resistance, which were all highly consistent with their adaptation of heat or cold habitat. Moreover, stress-related proteins were also accumulated in *P. lactiflora* Pall “Hangshao” seeds, showing its potential for stress resistance. Further protein analysis showed that the primary composition of PSP was albumin and globulin. And the solubility of PSP was good. Furthermore, PSP also showed high glucosidase inhibition activity, indicating that PSP might have some potential function for the remission of hyperglycemia. And *P. ostii* “Fengdan” seeds may be a better source for protein production than seeds of the other two species in terms of protein solubility and the content of total protein, albumin, and globulin. In addition, an optimal protocol of microwave-assisted alkali extraction was developed to produce PSP. In conclusion, the evaluated stress-related proteins in three peony seed species by proteomic analysis quite agreed with their adaptation of heat or cold stress; proteomics could also be a very useful tool for distinguishing species in the production; and peony seeds may be a good source for protein production.

## 1. Introduction

Peony is well known for its ornamental, medicinal, and edible properties, and it has become a commercial crop in China [1]. The economic benefit of peony is particularly increased by peony seed oil (PSO), which has more than 90% unsaturated fatty acids (UFAs) and more than 40% *α*-linolenic acid content [2]: *Paeonia ostii* “Fengdan” and *P. rockii* are the main species for the production of PSO [3, 4]. Moreover, high content of UFAs (more than 88.36%) is also found in *P. lactiflora* Pall “Hangshao” seeds [5]. In addition to seed oil, there are many other nutritional and bioactive compounds in peony seeds, such as protein, phenolic compounds, monoterpene glycosides, *γ*-tocopherol, and paeoniflorin, which could be used in the production of health products and medicines [6].

High plant-protein diets contribute to weight control and have a significantly beneficial effect on cardiovascular disease [7]. Peony seed meal (the by-product of oil extraction) has high protein content (32.44%) [2]. However, it is usually used as low value-added fertilizer or animal feed. It has been reported that PSP has favorable amino acid profiles and desirable physicochemical properties [2, 8]. The essential amino acids of PSP account for 39.62% ((*E*/*T*) essential amino acids to total amino acids), which is equal to soybean protein (*E*/*T*, 39.33%) [9] and higher than FAO/WHO-suggested value (*E*/*T*, 36.00%) [10]. The ratio (63.86%) of the aromatic amino acids (tyrosine and phenylalanine) to the branched-chain amino acids (isoleucine, leucine, and valine) in PSP is much higher than that in soybean protein (49.72%), suggesting that PSP could improve muscle metabolism and maintain protein homeostasis [11]. The low ratio (0.25) of lysine to arginine in PSP may positively affect arginine uptake, which might help to prevent cardiovascular, atherogenic, and lipidemic disease [12]. Moreover, high content (18.48%) of glutamic acid in PSP is useful for the regulation of glycogen synthesis and protein degradation, which is favorable for human health [13]. These results suggest that abundant amino acid composition in PSP plays an important role in regulating various physiological functions in the human body. Moreover, many *α*-glucosidase inhibitors, such as peptides, glycosides, flavonoids, alkaloids, and terpenoids, have also been isolated from plants, including peony [14]. Natural sources containing *α*-glucosidase inhibitors could be used to develop functional foods against diabetes. Therefore, the PSP could be used as a potential food ingredient with some beneficial functions and desirable properties [2].

Proteomic study is important for biological phenomena, developmental process, and metabolites synthesis. Some research on peony proteomics has been reported. Transcriptomic and proteomic analysis of *P. ostii* seeds was carried out to study the developmental stages and candidate genes related to oil biosynthesis and fatty acid metabolism [15]. Ren et al. [16] reported the mechanism of *Paeonia ostii* “Fengdan” seed germination as affected by low temperature using dynamic proteomic analysis. A quantitative proteomic analysis using isobaric tags for relative and absolute quantitation (iTRAQ) technology was performed on the stigma 24 h after pollination to better understand the molecular mechanisms involved in cross-incompatibility between tree peony (*Paeonia ostii* cv. Fengdanbai) and herbaceous peony (*Paeonia lactiflora* Pall. cv. Fenyunu) [17]. Zhao et al. [18] studied the mechanism of herbaceous peony in response to paclobutrazol inhibition of lateral branching using quantitative proteomics. Differential expression proteins have been studied during chilling treatment of tree peony (*Paeonia suffruticosa*) to illuminate the mechanism of bud dormancy release as affected by low temperature [19]. All those results show that proteomics is an important tool for the research of life sciences. Environmental factors in regional distribution or origin area greatly contribute to their differences in resistance [20]. *P. ostii* “Fengdan,” *P. rockii*, and *P. lactiflora* Pall “Hangshao” are potential oil plants. Among which, *P. ostii* “Fengdan” originated from South China, *P. rockii* originated from North China, and the distribution of *P. lactiflora* Pall “Hangshao” plants is extensive. Thus, it is widely agreed that *P. rockii* has high cold tolerance, *P. ostii* “Fengdan” has strong ability to resist heat, wet, and pathogens, while *P. lactiflora* Pall “Hangshao” also has high resistance to temperature and pathogen stress due to its extensive distribution. Proteomics may help to explain the mechanism of cold and heat stress tolerance in these three peony species. Moreover, proteomic analysis is also useful for germplasm resource identification [20, 21]. Gliadins, low molecular weight glutenin subunits (LMW-GS), and high molecular weight glutenin subunits (HMW-GS) are reliable genetic markers for investigating diversity of wheat germplasms and their relatives [22, 23]. The stress resistance potential, selling price, and seed oil composition of *P. ostii* “Fengdan,” *P. rockii*, and *P. lactiflora* Pall “Hangshao” are quite different [4]. Therefore, it is very important to make a germplasm resource identification system to avoid economic loss and uncertain quality problems caused by artificial adulteration. The current identification system of peony germplasms is focused on morphological characteristics, molecular markers [24], and microsatellites [25]. Proteomics has not been broadly used to study peony germplasms [8, 15–19]. Sodium dodecyl sulfate polyacrylamide gel electrophoresis (SDS-PAGE) and two-dimensional gel electrophoresis (2-DE) are two useful methods to separate and analyze proteins in proteomic research [20, 21].

In this study, the seed proteins from *P. ostii* “Fengdan,” *P. rockii*, and *P. lactiflora* Pall “Hangshao” were first extracted and identified. Then, seed protein profiles of the three peony species were analyzed. The relationship between the stress resistance potential and protein profiles of the three peony species was also investigated. Finally, the glucosidase inhibition activity of PSP was studied and the processing technology for protein production was also optimized based on alkali and microwave-assisted extraction methods. The results of this study are significant for germplasm resource identification and PSP product development.

## 2. Materials and Methods

### 2.1. Materials

Seeds of *P. ostii* “Fengdan,” *P. rockii*, and *P. lactiflora* Pall “Hangshao” were all collected from the peony germplasm resource nursery at the Institute of Vegetables and Flowers, Chinese Academy of Agricultural Sciences, in 2016. Seeds of *P. ostii* “Fengdan,” *P. rockii*, and *P. lactiflora* Pall “Hangshao” were collected from 30 plants at random, and fully mixed seeds were selected to do the experiment.

### 2.2. Measurement of Morphological Parameters and Nutrition Components of Seeds

A total of 90 seeds were separated into three groups and used to measure longitudinal and transverse diameters. Fresh weight was determined using 300 seeds with three repetitions. The water content, soluble sugar content, and starch content were measured according to the method of Ren et al. [16]. Crude protein content was determined according to the Chinese national standard for the determination of crude protein in oil plants (GB/T 14489.2-2008). Fatty acid was extracted and determined according to the method of Wei et al. [26].

### 2.3. Extraction and Separation of Albumins, Globulins, Gliadins, Glutenins, and Total Proteins in One-Way SDS-PAGE

Albumins, globulins, gliadins, and glutenins in one-way SDS-PAGE analysis were extracted using double-distilled water (ddH_2_O), 0.5 M sodium chloride (NaCl), 70% ethanol, or 0.1 M potassium hydroxide, respectively. Albumins were removed from extracts during globulin extraction, while albumins and globulins were removed during gliadin extraction. Albumins, globulins, and gliadins were removed during glutenin extraction. The SDS-PAGE analysis followed the instructions of Wang et al. [23]. Samples were loaded on SDS-PAGE with (b1, b3, b5, and b7) or without (b2, b4, b6, and b8) denaturation (65°C thermostatic water bath).

Total protein was extracted using a saturated phenol method by one-way SDS-PAGE analysis with some modification. Briefly, total proteins were extracted using the Tris-saturated phenol and a lysis buffer containing 0.5 M Tris-HCl (pH 7.5), 0.7 M sucrose, 0.1 M potassium chloride, 0.05 M ethylenediaminetetraacetic acid (EDTA), and 2% *β*-mercaptoethanol. The phenol phase was collected after centrifugation at 12,000 rpm (4°C) for 10 min and precipitated in 0.1 M ammonium acetate methanol solution (5 times the volume of the extracts) at -20°C for 12 hours. The protein precipitate was collected after centrifugation at 12,000 rpm (4°C) for 5 min and washed using precooled methanol and acetone. The protocol of SDS-PAGE analysis was according to the description of Wang et al. [23]. Samples were loaded on SDS-PAGE with (b1, b3, b5, and b7) or without (b2, b4, b6, and b8) denaturation.

### 2.4. Protein Extraction, Separation, and Identification in 2-DE Analysis

Protein separation and identification in 2-DE analysis were conducted as described before [16]. Total protein was extracted with buffer A (1 M pH 7.5 Tris-HCl, 0.1 M EDTA, 0.25 M sucrose, 1 mM PMSF, and 1 mM DTT) and buffer B (1 M pH 7.5 Tris-HCl, 0.1 M EDTA, 0.25 M sucrose, 4% Triton-100, 1 mM PMSF, and 1 mM DTT) according to the method described before [8, 27]. After centrifugation at 13,000 rpm and 4°C for 10 min, the supernatant was precipitated by 50% TCA buffer at -20°C for 3 hours. The precipitate was washed three times with chilled acetone containing DTT. The pellet was dried and dissolved in lysis buffer containing 7 M urea, 2 M thiourea, and 4% CHAPS. At last, protein concentrations were measured using a 2-D Quant Kit (Amersham Biosciences, USA) and BSA was used as the standard. Briefly, 5 *μ*l protein solution was mixed with 500 *μ*l precipitant and 500 *μ*l coprecipitant, respectively, and followed by a quick vortex. After centrifugation at 13,000 rpm for 5 min, the supernatant was mixed with 400 *μ*l ddH_2_O, 100 *μ*l copper solution, and the working color solution (colorA : colorB = 100 : 1). The fluorescence was measured at 480 nm, and protein concentration was calculated based on the BSA standard curve. The same amount of protein sample (600 *μ*g protein) from every peony species mixed with 0.5% IPG buffer, rehydration buffer (8 M urea, 2% CHAPS), 1% (*w*/*v*) DTT, and 1 *μ*l 1% bromophenol blue was loaded onto an the IEF linear IPG strips (pH 3-10NL, 18 cm, GE Healthcare) and rehydrated using an Ettan™ IPGphor™ II. Isoelectric focusing was performed under the following conditions: 300 V for 1 hour, 500 V for 1 hour, 1000 V for 1 hour, 3000 V for 1 hour, and finally run at 8000 V for 80 kV h. After focusing, the strips were equilibrated twice in equilibration solution, and then, proteins were separated on 12% SDS-polyacrylamide gels. Protein spots were stained using Coomassie brilliant blue (CBB, R-250/G-250) and scanned at 600 dpi with a BIO-RAD GS-800 scanner. Differentially expressed protein (DEP) spots were figured out based on statistically significant differences between samples from three peony species using Student's *t*-test (abundance variation of at least 2-fold, *p* < 0.05) by an ImageMaster™ 2D Platinum Software (Amersham Biosciences).

The spots of interest were excised from gels and subjected to a destaining solution (30% ACN with 100 mM NH_4_HCO_3_). The gel pieces were lyophilized and rehydrated in 50 mM NH_4_HCO_3_ solution (containing 50 ng trypsin). The peptides were then extracted twice using 0.1% trifluoroacetic acid (TFA) in 50% acetonitrile (ACN). The extracts were pooled together and lyophilized. Peptide mixtures were redissolved in 0.1% TFA and mixed with *α*-cyano-4-hydroxycinnamic acid in 30% ACN and 0.1% TFA before spotting onto the target plate. Protein identification was performed on an AB SCIEX MALDI TOF-TOF™ 5800 Analyzer (AB SCIEX, Foster City, CA, USA). Mass maps were acquired in positive ion reflector mode with 1000 laser shots per spectrum. The PMF peak detection criteria used were a minimum *S*/*N* of 10, local noise window width mass/charge (*m*/*z*) of 250, and minimum full-width half-maximum (bins) of 2.9. A maximum of 15 precursors per spot with a minimum signal/noise ratio of 50 were selected for MS/MS analysis. The contaminant *m*/*z* peaks were excluded from MS/MS analysis. An energy level of 2 kV was used for the collision-induced dissociation, with 3000 acquisitions accumulated for each MS/MS spectrum. A combined MS and MS/MS search was performed against the NCBI nr database for Malus. All automatic data analysis and database searches were conducted using GPS Explorer™ (ver. 3.6, AB SCIEX) running a Mascot search algorithm (v2.3, Matrix Science, London, UK) for protein identification. The raw MS and MS/MS spectra were processed using GPS Explorer™ software. The searches were conducted using the following settings: trypsin as the digestion enzyme, one missed cleavage, 100 ppm precursor tolerance, MS/MS ion tolerance of 0.6 Da, and methionine oxidation as the variable modification. Proteins with protein score confidenceintervals > 95% (proteinscore > 60) were considered as confident identifications. The identified proteins were then matched to specific processes or functions by searching Gene Ontology (http://www.geneontology.org/). Transcript information and chloroplast genomic data for *Paeonia* species are included in the plant genetic and proteomic bank. Our identified proteins were blasted and matched to these sequences in the plant genetic and proteomic bank.

### 2.5. Analysis of Protein Solubility and Glucosidase Inhibition Activity and Optimization of the Protein Extraction Process

Sample powder was fully mixed with distilled water to a solid-liquid ratio of 1 : 30. The temperature of the water bath was about 55°C, and pH was adjusted to 10.0. Alkaline protease with 10 times the volume of the protein powder was added. Enzymatic hydrolysis was stopped after 20 min, 40 min, 80 min, 150 min, and 400 min. Protein at different periods of enzymatic hydrolysis was fully mixed with distilled water and centrifuged at 5000 rpm for 20 min. The degree of protein hydrolysis was measured according to Nielsen et al. [28]. The nitrogen solubility index was equivalent to the amount of protein in the supernatant divided by the amount of protein in the stock solution.

About 5 g of sample powder was fully mixed with distilled water to a solid-liquid ratio of 1 : 35. Then, pH was adjusted to 10.5. Microwave power and time was set as 280 W and 150 s, respectively. About 10% alkaline protease was continuously added to the homogenized mixtures. Protein was hydrolyzed at 55°C for 3 hours with a pH of 10. The enzyme was inactivated at 100°C for 5 min. Supernatant was collected after centrifugation for further determination. The content of peptides was determined using the biuret method.

The inhibition rate of *α*-glucosidase was measured as follows. Samples, 0.05 M phosphate buffer solution (PBS), enzyme solution, and p-nitrophenyl-*α*-D-glucopyranoside (PNPG) (the detailed information is listed in Supplementary Table S1) were mixed together and reacted at 37°C in a water bath for 15 min. The reaction was terminated with a sodium carbonate solution. Absorbance was measured at 405 nm. The inhibition rate of *α*-glucosidase was determined according to the following formula. Inhibitionrate = (*A*_1_ − (*A*_2_ − *A*_4_))/(*A*_1_ − *A*_3_), where *A*_1_, *A*_2_, *A*_3_, and *A*_4_ were the absorbances corresponding to the control group, the measurement group, the blank group, and the background group, respectively.

To improve the protein extraction process, a single-factor experiment and an *L*_9_(3^4^) orthogonal experiment was carried out. Factors of the extraction process are shown in Supplementary Tables S2 and S3. No microwave treatment (microwave time is zero) was used as the control. Seeds were ground in a disintegrator (IKA, Guangzhou, China), and the powder was sieved using a 200-mesh sieve with a diameter of 450 *μ*m. The dry powder (10 g) was homogenized with deionized water (solid-liquid ratio (g/ml) ranging from 1 : 25 to 1 : 45), and pH was adjusted to 9.5-11.5. The supernatant was collected after centrifugation at 5000 rpm for 20 min. A microwave oven (Shunde, Guangdong, China) with 1000 W power was used in the extraction step. The supernatant was put in a microwave oven for 0-150 s at 210-490 W. Protein content was determined according to Ren et al. [16].

## 3. Results

### 3.1. Measurement of the Morphological Parameters and Physiological Characteristics of the Seeds of Three Peony Species

**Figure 1:**
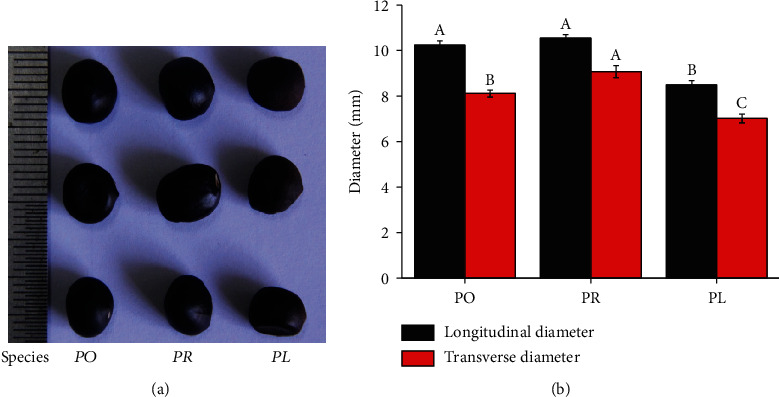
(a) Morphology and (b) longitudinal and transverse diameter of *P. ostii* “Fengdan” (PO), *P. rockii* (PR), and *P. lactiflora* Pall “Hangshao” (PL) seeds.

The seed surface of tree peony (*P. ostii* “Fengdan” and *P. rockii*) was very smooth, bright, and shiny black, and the shape of tree peony seeds was round and full, whereas the seed surface of herbaceous peony (*P. lactiflora* Pall “Hangshao”) was a little rough in dark brown and its shape was not very full (Figure 1(a)). The longitudinal diameter of three peony seeds was slightly longer than the transverse diameter, and the longitudinal to transverse diameter ratio was about 1.2 (Figure 1(b)). The transverse diameter of *P. rockii* seeds was around 9.0 mm, the transverse diameter of *P. ostii* “Fengdan” seeds was about 8.0 mm, and the transverse diameter of *P. lactiflora* Pall “Hangshao” seeds was approximately 7.0 mm (Figure 1(b)). The longitudinal diameter of *P. rockii* and *P. ostii* “Fengdan” seeds was about 10 mm, which was significantly bigger than that of *P. lactiflora* Pall “Hangshao” seeds (8 mm) (Figure 1(b)). Water content was increased in *P. ostii* “Fengdan” seeds (8.71%) compared to *P. rockii* (8.21%) and *P. lactiflora* Pall “Hangshao” seeds (8.08%) (Table 1). Biomass (fresh weight) of *P. rockii* seeds (0.30 g) was the greatest, and *P. ostii* “Fengdan” seeds (0.26 g) also increased in biomass over *P. lactiflora* Pall “Hangshao” seeds (0.21 g) (Table 1).

Soluble sugar content was the highest in *P. lactiflora* Pall “Hangshao” seeds (10.87%), followed by *P. rockii* seeds (9.49%), and it was the lowest in *P. ostii* “Fengdan” seeds (8.61%) (Table 1). Starch content was enhanced in *P. lactiflora* Pall “Hangshao” (10.46%) and *P. rockii* seeds (10.16) over that of *P. ostii* “Fengdan” seeds (7.44%). There was much more protein in *P. ostii* “Fengdan” seeds (20.7%) compared with *P. rockii* (18.9%) and *P. lactiflora* Pall “Hangshao” (16.5%) seeds. Total fatty acid content was significantly higher in *P. lactiflora* Pall “Hangshao” seeds (16.8%) than in *P. rockii* (11.5%) and *P. ostii* “Fengdan” (10.3%) seeds. And protein content was the greatest among four basic nutrient compositions.

### 3.2. Analysis of Albumins, Globulins, Gliadins, Glutenins, and Total Proteins Using One-Way SDS-PAGE

**Figure 2:**
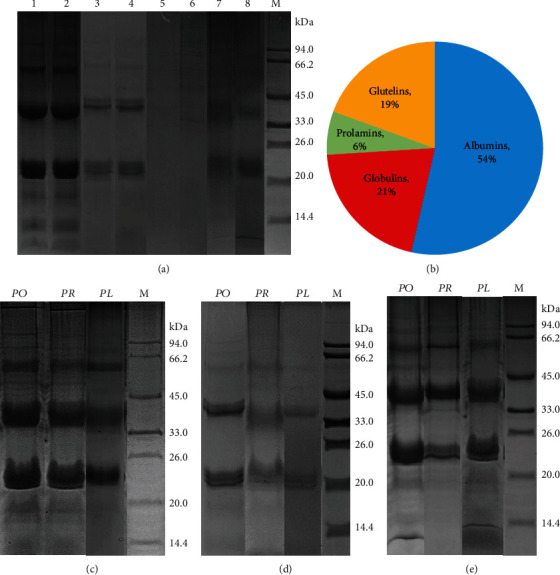
(a) Comparison of albumin, globulin, gliadin, and glutenin pretreatment with or without 65°C denaturation using SDS-PAGE analysis, (b) ratio of these protein fractions to total proteins, and SDS-PAGE analysis of (c) albumins, (d) globulins, and (e) total proteins extracted from seeds of three peony species. a-1: albumin, 2-ME/SDS buffer, 65°C water bath extraction, and 65°C denaturation; a-2: albumin, 2-ME/SDS buffer, 65°C water bath extraction, and room temperature; a-3: globulin, 2-ME/SDS buffer, 65°C water bath extraction, and 65°C denaturation; a-4: globulin, 2-ME/SDS buffer, 65°C water bath extraction, and room temperature; a-5: gliadin, 2-ME/SDS buffer, 65°C water bath extraction, and 65°C denaturation; a-6: gliadin, 2-ME/SDS buffer, 65°C water bath extraction, and room temperature; a-7: glutelin, 2-ME/SDS buffer, 65°C water bath extraction, and 65°C denaturation; and a-8: glutelin, 2-ME/SDS buffer, 65°C water bath extraction, and room temperature. M: protein marker.

Albumins, globulins, gliadins, and glutenins with or without 65°C denaturation pretreatment were analyzed by one-way SDS-PAGE (Figure 2(a)). The bands of albumins, globulins, and glutenins were somewhat improved using 65°C denaturation, suggesting that a pretreatment of 65°C denaturation should be used for SDS-PAGE analysis. The size of albumins, globulins, and glutenins was about 8-120 kDa, 20-94 kDa, and 10-94 kDa, respectively. The number of albumin bands was the most, the color was the darkest, and the bands were clearest (Figure 2(a)); the bands of globulins were also very clear, but at a weaker intensity than those of albumins (Figure 2(a)); and the bands of gliadins and glutenins were very blurry, especially those of gliadins (Figure 2(a)). Consistently, the content of albumins was the highest (at 54% of the total), followed by globulins (21%) and glutenins (19%), and the content of gliadins was the lowest (6%) in *P. ostii* “Fengdan” seeds (Figure 2(b)).

The albumin, globulin, and total protein separation in *P. ostii* “Fengdan,” *P. rockii*, and *P. lactiflora* Pall “Hangshao” seeds is shown in Figures 2(c)–2(e). The size of albumins was about 8-120 kDa in *P. ostii* “Fengdan” and *P. rockii* seeds, while it ranged from 20 to 94 kDa in *P. lactiflora* Pall “Hangshao” seeds (Figure 2(c)). The number of albumin bands was the greatest in *P. ostii* “Fengdan” seeds, followed by *P. rockii* seeds, and there were only three bands in *P. lactiflora* Pall “Hangshao” seeds. The size of globulins was about 20-94 kDa in *P. ostii* “Fengdan” and *P. rockii* seeds, while it was 20 to 40 kDa in *P. lactiflora* Pall “Hangshao” seeds (Figure 2(d)). The number and intensity of globulin bands were the highest in *P. ostii* “Fengdan” seeds, followed by *P. rockii* seeds, and they were the lowest in *P. lactiflora* Pall “Hangshao” seeds. There were only two blurry globulin bands (about 20 kDa and 40 kDa) in *P. lactiflora* Pall “Hangshao” seeds. The size of total protein bands in *P. ostii* “Fengdan,” *P. rockii*, and *P. lactiflora* Pall “Hangshao” seeds was about 8-100 kDa, 23-94 kDa, and 14-66 kDa, respectively (Figure 2(e)). The greatest number and highest intensity of total protein bands were also found in *P. ostii* “Fengdan” seeds, followed by *P. lactiflora* Pall “Hangshao” seeds. Overall, the primary proteins were albumin and globulin in peony seed, and the greatest content of albumin, globulin, and total protein was all found in *P. ostii* “Fengdan” seeds.

### 3.3. Protein Identification and Expression Analysis

**Figure 3:**
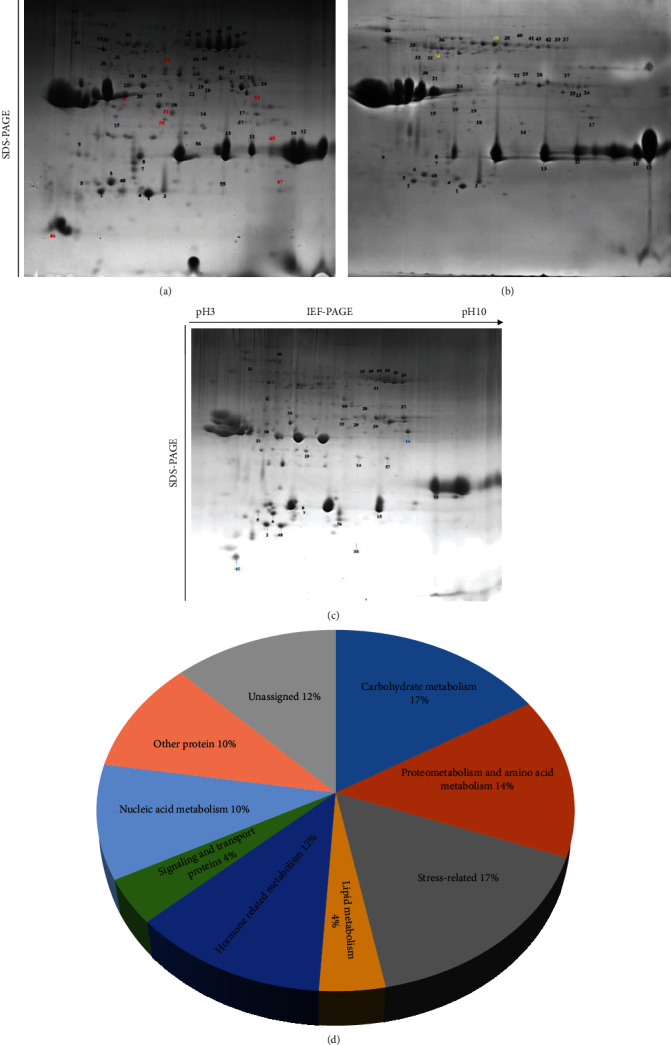
Proteome maps of total proteins from (a) *P. ostii* “Fengdan,” (b) *P. rockii*, and (c) *P. lactiflora* Pall “Hangshao” seeds and (d) gene ontology analysis of differentially expressed proteins (DEPs). Same amount of protein sample (600 *μ*g protein) from every peony seed species was loaded onto the IEF linear IPG strips to make sure that same amount of protein was analyzed. The SEPs in *P. ostii* “Fengdan”, *P. rockii*, and *P. lactiflora* Pall seeds are marked with red, yellow, and blue, respectively.

**Figure 4:**
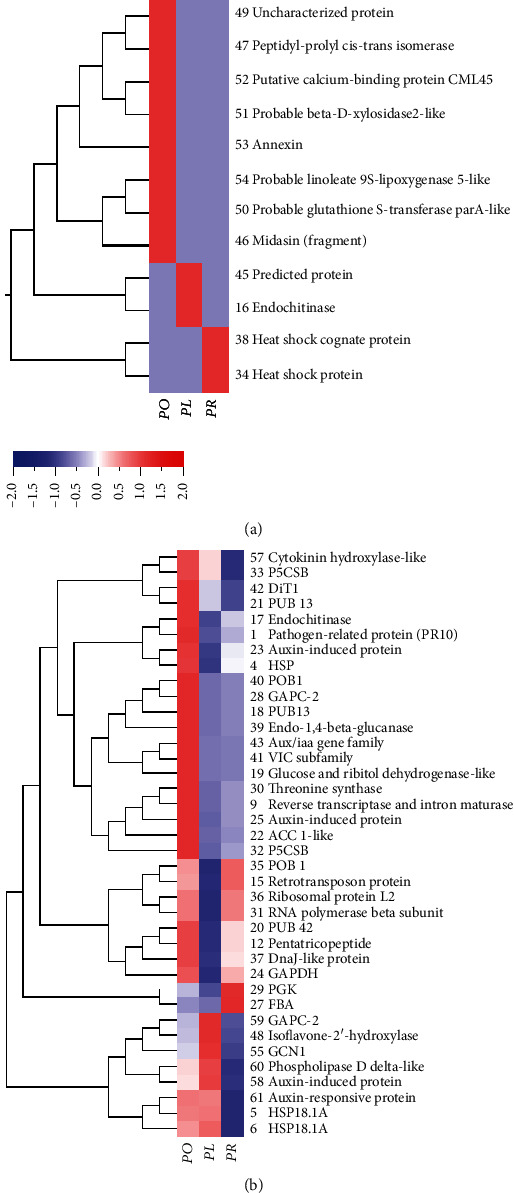
Hierarchical clustering analysis of (a) specifically expressed proteins (SEPs) and (b) DEPs of three peony species. Abbreviations: P5CSB: delta 1-pyrroline-5-carboxylate synthetase B; DiT1: 2-oxoglutarate/malate translocator; PUB13: U-box domain-containing protein 13; PR10: pathogen-related protein; HPS: heat shock protein; POB1: POZ/BTB-containing G-protein 1 isoform 1; GAPC-2: glyceraldehyde-3-phosphate dehydrogenase 2; PUB13: U-box domain-containing protein 13; VIC: voltage-gated ion channel superfamily; ACC 1-like: acetyl-CoA carboxylase 1-like; PUB 42: U-box domain-containing protein 42-like; GAPDH: glyceraldehyde-3-phosphate dehydrogenase; PGK: phosphoglycerate kinase; FBA: fructose-bisphosphate aldolase, cytoplasmic isozyme; GCN1: eIF-2-alpha kinase activator; HSP18.1A: heat shock protein 18.1A.

The specifically expressed proteins (SEPs) and differentially expressed proteins (DEPs) are annotated in Figures 3(a)–3(c) with different colors. A total of 61 proteins were identified from *P. ostii* “Fengdan,” *P. rockii*, and *P. lactiflora* Pall “Hangshao” seeds (Table 2). Among these 61 proteins, eight, two, and two SEPs were only identified from *P. ostii* “Fengdan,” *P. rockii*, and *P. lactiflora* Pall “Hangshao” seeds, respectively. And there were 49 DEPs shared among the seeds of all three species. All those proteins were widely distributed in a range of pH 3-10. Gene Ontology analysis were assigned to DEPs and divided into nine groups, including carbohydrate metabolism (17%), proteometabolism and amino acid metabolism (14%), lipid metabolism (4%), stress related (17%), hormone-related metabolism (12%), signaling and transport proteins (4%), nucleic acid metabolism (10%), other proteins (10%), and unassigned proteins (12%) (Figure 3(d)). Two SEPs (34 and 38) identified from *P. rockii* seeds were both stress related (Figure 4(a)). One SEP (16) identified from *P. lactiflora* Pall “Hangshao” seeds was carbohydrate metabolism related and the other (45) one from *P. lactiflora* Pall “Hangshao” seeds was unassigned (Figure 4(a)). Among the eight SEPs identified from *P. ostii* “Fengdan” seeds, two (47 and 50) were proteometabolism and amino acid metabolism related, two (46 and 52) were nucleic acid metabolism related, one (51) was carbohydrate metabolism related, one (53) was stress related, one (54) was lipid metabolism related, and one (49) was unassigned (Figure 4(a)).

The expression patterns of the 38 important DEPs were divided into three clusters: (1) Cluster I, including 28 DEPs that were highly enhanced in *P. ostii* “Fengdan” seeds, most of these proteins (85.7%) were the lowest in *P. lactiflora* Pall “Hangshao” seeds; (2) Cluster II, including two DEPs that were the highest in *P. rockii* seeds, followed by *P. ostii* “Fengdan” seeds; and (3) Cluster III, including eight DEPs that were highly accumulated in *P. lactiflora* Pall “Hangshao” seeds, followed by *P. ostii* “Fengdan” seeds (Figure 4(b)).

### 3.4. Analysis of Protein Solubility

**Figure 5:**
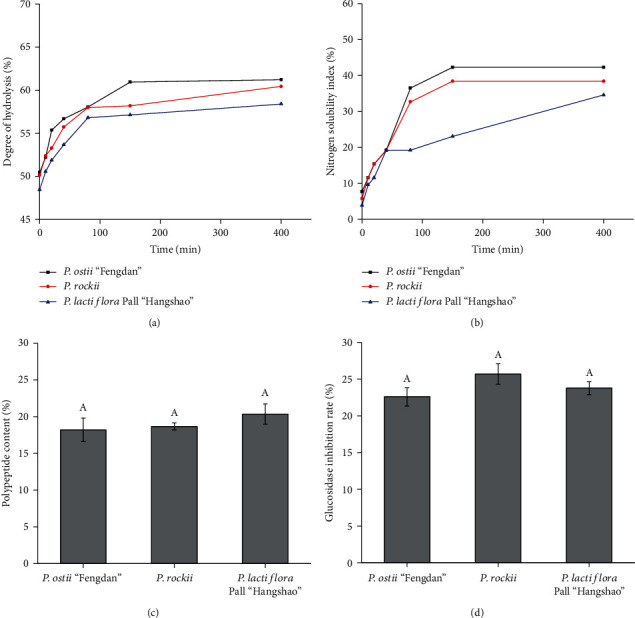
(a) Degree of hydrolysis, (b) nitrogen solubility index, (c) peptide content, and (d) glucosidase inhibition rate of proteins extracted from *P. ostii* “Fengdan,” *P. rockii*, and *P. lactiflora* Pall “Hangshao” seeds. Significant separation within treatments was assayed by an analysis of variance (ANOVA) and Duncan's multiple range tests at the 5% level.

The degree of protein hydrolysis and solubility were determined at 0, 10, 20, 40, 80, 150, and 400 min after hydrolysis. In general, both degree of protein hydrolysis and nitrogen solubility index (NSI) were the highest in proteins extracted from *P. ostii* “Fengdan” seeds, followed by those extracted from *P. rockii* seeds (Figures 5(a) and 5(b)). After hydrolysis for 400 min, the hydrolysis degree of PSP ranged between 58.4% and 61.2%, and NSI of PSP was about 34.6%-42.3%.

### 3.5. Analysis of Glucosidase Inhibition Activity

To verify the glucosidase inhibition activity of PSP, peptides were extracted from seeds of *P. ostii* “Fengdan,” *P. rockii*, and *P. lactiflora* Pall “Hangshao,” and the content of peptide and their glucosidase inhibition ratios were estimated. Results showed that the activity of *α*-glucosidase was reduced remarkably by the addition of peony seed peptides. The peptide content in seed protein of three peony species was 18.2%-20.4%, while the glucosidase inhibition rate was 22.6%-25.7% (Figures 5(c) and 5(d)). Peptide content and glucosidase inhibition rate were not significantly different among the three peony species. Based on the degree of hydrolysis, protein solubility, and glucosidase inhibition rate, *P. ostii* “Fengdan” seeds were a better choice for developing high-value protein products with good hydrolysis and dissolution characteristics and a desirable glucosidase inhibition activity.

### 3.6. Optimization of the Protein Extraction Process

**Figure 6:**
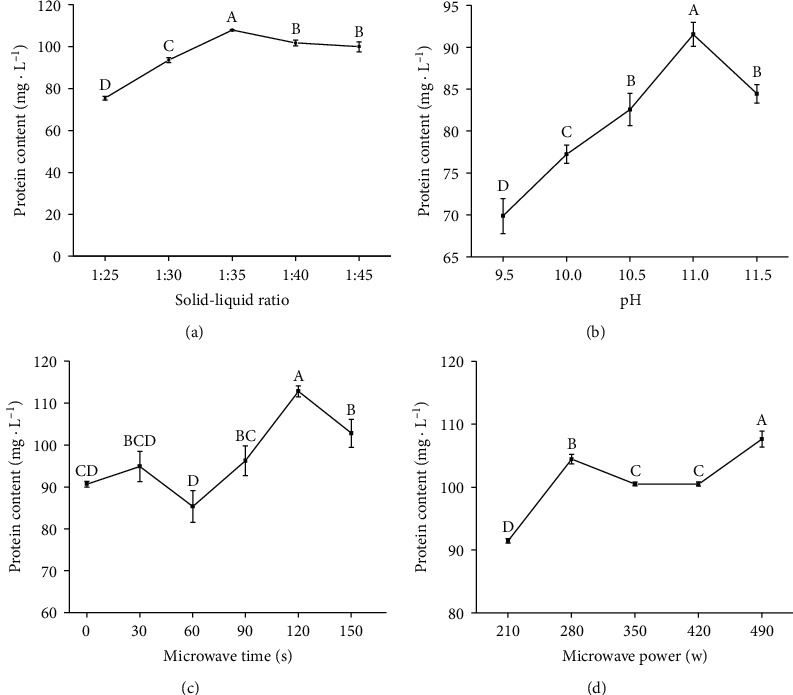
Protein content as affected by (a) solid-liquid ratio, (b) pH, (c) microwave time, and (d) microwave power in a single-factor experiment.

A single-factor experiment and an *L*_9_(3^4^) orthogonal methodology experiment were carried out to optimize the protein extraction process (Supplementary Tables S2 and S3). The extraction solid-liquid ratio, pH, microwave time, and microwave power had great effect on protein extraction efficiency. Protein extraction efficiency was first increased as the solid-liquid ratio increased and peaked at a solid-liquid ratio of 1 : 35 (Figure 6(a)). After that, it decreased with an increase in the solid-liquid ratio. For the pH of the protein extraction process, protein content increased from pH 9.5 to pH 11.0 and then decreased when pH was higher than 11.0 (Figure 6(b)). Microwave-assisted protein extraction significantly increased the protein content. Protein extraction efficiency was the greatest with 120 s of microwaving (Figure 6(c)). For microwave power, protein content increased first, then decreased, and finally increased again to reach a maximum at 490 W (Figure 6(d)). Above all, the single-factor experiment showed that protein extraction efficiency was the greatest with a solid-liquid ratio of 1 : 35, a pH of 11.0, and 120 s of microwaving at 490 W. Moreover, the results of the *L*_9_(3^4^) orthogonal methodology experiment showed that the optimum conditions were solid-liquid ratio of 1 : 35, microwave power at 280, microwave time of 120 s, and pH at 10.5 (Table 3).

## 4. Discussion

Recently, the components of peony seeds have become a focus of attention. The high content of unsaturated fatty acids (UFAs), protein, and secondary metabolites found in the peony seeds may greatly contribute to their favorable medicinal and nutritional characteristics [1, 2, 16]. PSP is considered to be a potential food ingredient due to its satisfactory amino acid composition and beneficial functions [2]. Moreover, proteomics is an important tool for the study of biological phenomena and germplasm resource identification. Quality problems caused by artificial adulteration of seeds were becoming serious in commercial peony seed production and their processed products. Therefore, it is necessary to establish peony germplasm identification methods, study the protein profiles, and evaluate the physicochemical and functional characteristics of the extracted PSP.

### 4.1. Analysis of Basic Nutrient Composition of Seeds

Seeds are important storage organs in nature that provide nutrition for embryo development and germination. Seeds of angiosperms are always rich in oil, starch, and protein. Four kinds of basic nutrient components, including proteins, starch, soluble sugar, and fatty acids, have been detected in peony seeds. Of these, crude protein content was the highest, which was significantly higher than the content of fatty acids and starch. The nutritional compositions of peony seeds were consistent with *Cucurbita maxima* seeds [29]. The highest level of crude protein was detected in *P. ostii* “Fengdan” seeds, followed by *P. rockii* seeds. Encouragingly, seed protein content from all these three peony species was greater than that from wheat seeds, the leading source of vegetal protein in human food [30], suggesting the advantages and potential of peony seeds, especially for *P. ostii* “Fengdan” seeds, in protein product development.

### 4.2. Peony Germplasm Resource Identification by One-Way SDS-PAGE and SEPs in 2-DE

Accurate and rapid laboratory techniques for germplasm resource identification are becoming increasingly important for protecting the rights and interests of consumers and ensuring quality and safety of commercial food product. There is always artificial adulteration of peony seeds (most commonly, mixed seeds from different peony species) on the peony market due to rough production methods and profit-driven system, which greatly reduced the quality of seed products, such as peony seed oil and PSP. To identify and avoid artificial adulteration of peony seeds, we developed two strategies: SDS-PAGE could distinguish the mixed tree and herbaceous peony seeds effectively; SEPs in 2-DE could be used as protein markers to identify seeds of these three peony species on the market. In general, tree (*P. ostii* “Fengdan” and *P. rockii*) and herbaceous (*P. lactiflora* Pall “Hangshao”) peony seeds could be roughly distinguished by color and seed size (Figure 1), which should be verified by scientific technology method. In this study, SDS-PAGE was effective to distinguish the mixed tree and herbaceous peony seeds: the bands of albumins and globulins were much more obvious in *P. ostii* “Fengdan” and *P. rockii* seeds than in *P. lactiflora* Pall “Hangshao” seeds (Figure 2). Moreover, protein markers developed from 2-DE-based proteomics are widely used in plants to assess genetic variability [31]. A 2-DE-based dynamic proteomic technique could be used to distinguish *P. ostii* “Fengdan” and *P. rockii* seeds. Most SEPs were highly expressed in *P. ostii* “Fengdan” seeds, and the number of SEPs was also the greatest in *P. ostii* “Fengdan” seeds. All in all, seeds of *P. ostii* “Fengdan,” *P. rockii*, and *P. lactiflora* Pall “Hangshao” can be easily distinguished by SEPs: there were eight SEPs (46, 47, 49, 50, 51, 52, 53, and 54) in *P. ostii* “Fengdan” seeds, two SEPs (34 and 38) in *P. rockii* seeds, and two SEPs (16 and 45) in *P. lactiflora* Pall “Hangshao” seeds. Those SEPs could be used as protein markers to identify seeds of these three peony species on the market and in the production. Other proteins, such as LMW-GS and HMW-GS are also developed as reliable genetic markers for investigating diversity of wheat germplasms [22, 23]. Therefore, SDS-PAGE and 2-DE technique could be very useful tools to distinguish mixed seeds from different species and then avoid artificial adulteration and protect the interests of consumers.

### 4.3. Peony Seed Protein Profiles and Stress Resistance Analysis of Three Species

*P. ostii* “Fengdan,” *P. rockii*, and *P. lactiflora* Pall “Hangshao” are important species that are used to produce peony seed oil [3, 5]. In China, *P. ostii* “Fengdan” is mainly distributed in Anhui, Henan, Hubei, Shaanxi, and Sichuan provinces and *P. rockii* mainly grows in Gansu, Shaanxi, and Hubei provinces [32], while the distribution of *P. lactiflora* Pall “Hangshao” plants is extensive. Environmental factors in regional distribution greatly contribute to plant growth characteristics [8]. Therefore, it is widely accepted that *P. rockii* has high cold tolerance, *P. ostii* “Fengdan” has strong ability to resist heat, wet, and pathogens, and *P. lactiflora* Pall “Hangshao” also has high resistance to temperature and pathogen stress in many years of peony production. Heat shock proteins (HSPs) respond to stressful conditions, such as cold and oxidative stress [33]. As a molecular chaperone, DnaJ-like protein specifically regulates HSP70 [34]. Two heat shock proteins (34 and 38) were found only in *P. rockii* seeds, indicating that it has high cold tolerance, which was consistent with its geographical distribution and stress resistance potential. Moreover, two DEPs (Nos. 27 and 29) belonging to carbohydrate metabolism (100%) were highly expressed in *P. rockii* seeds, resulting in strong carbohydrate metabolism. Soluble sugars, the products of carbohydrate metabolism, have been proved to enhance cold tolerance [35], which could be another supporting evidence for the high cold tolerance of *P. rockii.*

Delta 1-pyrroline-5-carboxylate synthetase (P5CS) is essential in proline biosynthesis and osmoregulation in plants [36]. Highly accumulated P5CS (Nos. 32 and 33), pathogen-related (No. 1), HSP (No. 4), and DnaJ-like proteins (No. 37) in *P. ostii* “Fengdan” seeds supported its strong ability to resist heat and pathogen stress. Moreover, there were eight SEPs in seeds of *P. ostii* “Fengdan” and five of them are stress related. Peptidyl-prolyl *cis*-*trans* isomerase (PPIase, No. 47) is involved in thermotolerance [37]. Glutathione S-transferase (GSTs, No. 50) is an evolutionarily conserved enzyme that is important in the detoxification of many xenobiotic compounds and is involved in oxidative stress [38]. Calcium is an important signaling molecule that can respond to various stress signals. Calcium-binding protein 45 (CML45, No. 52) plays an important role in insect, disease, and pathogen resistance [39]. Annexin I (No. 53) is a stress protein induced by heat, oxidative stress, and a sulfhydryl-reactive agent [40]. Oxylipins from the linoleate 9S-lipoxygenase (9-LOX, No. 54) pathway function in lateral root development and pathogen arrest [41]. To summarize briefly, the accumulated PPIase (No. 47) and annexin I (No. 53) in *P. ostii* “Fengdan” seeds may enhance its thermotolerance, while the enhanced CML45 (No. 52) and 9-LOX (No. 54) indicate its strong ability to resist pathogens and diseases.

One SEP of endochitinase was found in *P. lactiflora* Pall “Hangshao” seeds. Endochitinase may be associated with antagonistic activity against phytopathogenic fungi [42]. Thereby, it was deduced that *P. lactiflora* Pall “Hangshao” had high fungi stress tolerance. Eight DEPs were highly expressed in *P. lactiflora* Pall “Hangshao” seeds including one isoflavone 2′-hydroxylase (No. 48), two HSPs (Nos. 5 and 6), one GAPC-2 (No. 59), one phospholipase D delta (No. 60, involved in the nonhost resistance and basal defense) [43], and two auxin-related proteins (Nos. 58 and 61). In conclusion, the elevated accumulations of stress-related proteins in *P. lactiflora* Pall “Hangshao” seeds showed its high resistance to temperature stress and pathogens.

### 4.4. Peony Seed Protein Characteristics, Function, and Extraction

Based on the Osborne classification (solubility in various solvents), proteins are divided into four groups: albumins, globulins, prolamins, and glutelins. Albumins and globulins are often functional enzymes, while glutelins and prolamins are storage proteins with different amino acid compositions [44]. Albumins and globulins account for 25% of total wheat seed proteins, while prolamins and glutelins are about 75% of the total [30]. Unlike protein composition in wheat seeds, albumins and globulins were dominant (75%) in peony seeds, and its storage protein (prolamins and glutelins) content was about 25% of total proteins. This property (low storage protein content) of PSP is similar to pulse crops [45] and complementary to wheat seed proteins [30]. These differences may be caused by species specificity, seed structure, and metabolic diversity. A combination of peony, bean, and wheat protein may provide proper, essential, and relatively complete nutrition for humans. Plant proteins facilitate weight control, reduce blood pressure and cholesterol, and inhibit heart disease, stroke, and cancer [7]. Albumins and globulins also have some positive effects on human health due to their free radical-scavenging activity [46]. High levels of albumins and globulins in peony seeds, especially in *P. ostii* “Fengdan” seeds, showed their antioxidant capacity and potential medicinal values. Isoflavone 2′-hydroxylase participates in isoflavonoid biosynthesis. Isoflavones in soy protein have a beneficial role in obesity [47]. Evaluated isoflavone 2′-hydroxylase was also found in peony seeds, indicating that PSP might also be helpful for alleviating obesity.

Glucose produced is always absorbed into the blood, resulting in the elevated blood sugar. Therefore, *α*-glucosidase is closely related with hyperglycemia. Many serious diseases are late complications of hyperglycemia, threatening people's health and life. *α*-Glucosidase can hydrolyze the cleavage of glucose from disaccharides and oligosaccharides. Inhibition of *α*-glucosidase activity can prevent these late complications by decreasing the postprandial rise in blood glucose [48]. Synthetic medicines, such as metformin, and acarbose are efficient *α*-glucosidase inhibitors, but they also have serious side effects, like adverse gastrointestinal symptoms and hepatotoxicity [49, 50]. Natural peptides, degradation products of natural proteins, are safe products with many biological activities including regulating blood glucose, protecting the liver, and immunoregulation [51]. *In vitro* hypoglycemic tests showed that PSP effectively inhibited the activity of *α*-glucosidase. The glucosidase inhibition rate of PSP was about 22.6%-25.7% without significant differences among three peony species in the present study. Peptides extracted from *Gypsophila oldhamiana* and *Momordica charantia* also have *α*-glucosidase inhibitory effects [51, 52]. The oral glucose tolerance test showed peptides from *M. charantia* produced a reduction of 25.50%, 39.62%, and 41.74% in blood glucose levels after 1, 2, and 3 hours, respectively [51]. Similar with peptides from *G. oldhamiana* and *M. charantia*, PSP peptides showed potent inhibition of *α*-glucosidase as expected. Conclusively, seed protein from all three peony species could potentially be applied in the food industry due to the favorable glucosidase inhibition activity.

Protein solubility is a vital parameter affecting the functionality and applicability of proteins [53]. The hydrolysis degree and protein solubility were the greatest in *P. ostii* “Fengdan” seeds, followed by *P. rockii* seeds, and the lowest in *P. lactiflora* Pall “Hangshao” seeds. Solubility of a protein product purified from *Brassica napus* was about 21.5% [53]. Solubility of PSP was about 40% with a 60% degree of hydrolysis. All in all, results showed that PSP could be used as a food product with some beneficial functions and desirable physicochemical properties. Moreover, *P. ostii* “Fengdan” seeds are a better source for protein production than seeds of the other two species in terms of hydrolysis degree and protein solubility.

For high-quality PSP production, an appropriate, harmless, and reliable processing technology needs to be developed. Microwave-assisted extraction can increase protein yield [54]. We tried to combine microwave-assisted extraction and the alkali extraction and acid precipitation method together. Methods and conditions of protein extraction greatly affected the yield and protein characteristics. Based on a single-factor experiment and an *L*_9_(3^4^) orthogonal experiment, an optimal protocol (solid-liquid ratio at 1 : 35, microwave power at 280, microwave time 120 s, and pH at 10.5) was developed, which is useful for the development of PSP.

## 5. Conclusions

In the present study, the physiological substance and protein profiles in seeds of *P. ostii* “Fengdan,” *P. rockii*, and *P. lactiflora* Pall “Hangshao” were analyzed. A combination of one-way SDS-PAGE and 2-DE could be used to distinguish seeds of *P. lactiflora* Pall “Hangshao,” *P. ostii* “Fengdan,” and *P. rockii*. The albumin and globulin content was the greatest in *P. ostii* “Fengdan” seeds, followed by *P. rockii* seeds. Moreover, proteomic analysis by 2-DE showed that the stress resistance potential of three peony species was related to those seed protein profiles, including HSP and thermotolerance-, stress-, and pathogen-related proteins. An *in vitro* test showed that PSP had high glucosidase inhibition activity. Above all, the seed proteomic profiles of three peony species supported their respective stress resistance characteristics and were very helpful for species identification. In addition, the PSP content was high in peony seed and the primary composition was albumin and globulin with good solubility and some potential function for the remission of hyperglycemia. Moreover, an optimal protocol of microwave-assisted alkali extraction was developed to produce PSP.

## Figures and Tables

**Table 1:** Morphological and physiological parameters of *Paeonia ostii* “Fengdan,” *P. rockii*, and *P. lactiflora* Pall “Hangshao” seeds.

Species	Water content (%)	Fresh weight (g)	Soluble sugar content (%)	Starch content (%)	Protein content (%)	Total fatty acid content (%)
*P. ostii* “Fengdan”	8.71 ± 0.02 a	0.26 ± 0.005 b	8.61 ± 0.11 c	7.44 ± 0.07 b	20.7 ± 0.43 a	10.3 b
*P. rockii*	8.21 ± 0.12 b	0.30 ± 0.003 a	9.49 ± 0.05 b	10.16 ± 0.28 a	18.9 ± 0.30 b	11.5 b
*P. lactiflora* Pall “Hangshao”	8.08 ± 0.07 b	0.21 ± 0.002 c	10.87 ± 0.28 a	10.46 ± 0.15 a	16.5 ± 0.47 c	16.8 a

Significant separation within treatments was assayed by an analysis of variance (ANOVA) and Duncan's multiple range tests at the 5% level.

**Table 2:** Identified proteins from *P. ostii* “Fengdan,” *P. rockii*, and *P. lactiflora* Pall “Hangshao” seeds.

Match ID		Protein name	Species	Accession no.	Matches	Protein score	Mr(expt)	Mr(calc)	ppm
1		Pathogen-related protein (PR10)	*Tanacetum cinerariifolium*	gi|342219047	6	97	718.2225	718.3796	-28.71
2	Uncharacterized protein	*Monoraphidium neglectum*	gi|926789281	7	87	791.2097	791.3297	-31.42
3		Uncharacterized protein	*Zea mays*	gi|194699792	19	84	792.2292	792.4204	-41.31
4		17.6 kDa class II heat shock protein	*Brassica napus*	gi|157849708	5	149	757.2159	757.4334	-27.23
5		Heat shock protein (HSP) 18. 1A	*Citrullus lanatus*	gi|315932718	13	197	712.2567	712.4119	-38.42
6		(ACC 1-like)	*Citrullus lanatus*	gi|315932718	11	176	712.2454	712.4119	-23.35
7		Putative uncharacterized protein	*Chlorella variabilis*	gi|307110140	20	77	767.3677	767.3636	5.29
8		Uncharacterized protein	*Marchantia polymorpha* subsp. *polymorpha*	gi|1026775669	13	79	762.2643	762.3806	-28.42
9		Putative reverse transcriptase and intron maturase	*Chara vulgaris*	gi|38638315	17	82	803.4559	803.4389	21.2
10		Putative uncharacterized protein OJ1076_H08.10	*Oryza sativa subsp. japonica*	gi|48843763	19	64	701.2101	701.3457	-43.25
11		Cytochrome P450	*Cynara cardunculus var. scolymus*	gi|976915107	12	68	794.2898	794.3229	-41.57
12		Pentatricopeptide repeat-containing protein	*Ananas comosus*	gi|1035954643	17	68	723.2332	723.3585	-39.34
13		Uncharacterized protein	*Zea mays*	gi|224035303	8	69	794.2876	794.436	-18.48
14		Pheophorbide A oxygenase, putative	*Ricinus communis*	gi|223533590	16	76	702.2987	702.4276	-33.34
15		Retrotransposon protein, putative	*Oryza sativa Japonica group*	gi|77554901	36	76	706.443	706.365	57.24
16		Endochitinase	*Medicago truncatula*	gi|355523213	5	103	796.3046	796.4344	-16.12
17		Endochitinase	*Medicago truncatula*	gi|355523213	4	98	1474.5738	1474.6325	-13.41
18		U-box domain-containing protein 13 (PUB13)	*Vitis vinifera*	gi|225448505	13	76	734.4143	734.3633	39.64
19		Glucose and ribitol dehydrogenase-like	*Glycine max*	gi|356571142	4	92	819.4404	819.4404	-24.34
20		U-box domain-containing protein 42-like (PUB 42)	*Solanum lycopersicum*	gi|460404308	22	88	719.3777	719.4251	-65.98
21		U-box domain-containing protein 13 (PUB13)	*Vitis vinifera*	gi|225448505	13	76	734.4143	734.3633	19.71
22		Acetyl-CoA carboxylase 1-like	*Vitis vinifera*	gi|225459364	31	79	727.5259	727.4956	41.7
23		Auxin-induced protein	*Helianthus annuus*	gi|2606077	12	253	776.3838	776.3817	2.67
24		Glyceraldehyde-3-phosphate dehydrogenase (GAPDH)	*Eleutherococcus senticosus*	gi|484849356	13	280	826.3752	826.4007	-30.92
25		Auxin-induced protein	*Helianthus annuus*	gi|2606077	7	243	776.3516	776.3817	-32.94
26		S-adenosyl-L-methionine-dependent methyltransferases superfamily protein, putative	*Theobroma cacao*	gi|508728070	14	60	849.4994	849.4266	44.62
27		Fructose-bisphosphate aldolase (FBA) cytoplasmic isozyme	*Vitis vinifera*	gi|225440976	8	119	1072.5656	1072.5124	29.53
28		GAPC-2	*Arabidopsis lyrata subsp. lyrata*	gi|297338591	17	323	810.3871	810.4058	-23.04
29		Phosphoglycerate kinase (PGK), partial	*Clermontia arborescens subsp. waihiae*	gi|428230938	6	331	1836.1179	1836.0356	14.77
30		Threonine synthase, chloroplastic-like	*Solanum lycopersicum*	gi|460392573	17	69	699.3353	699.3915	-50.02
31		RNA polymerase beta subunit	*Pinus rigida*	gi|198279315	1	52	1046.4547	1046.4637	-8.61
32		Delta 1-pyrroline-5-carboxylate synthetase B (P5CSB)	*Brassica napus*	gi|12667251	22	90	734.3891	734.3745	19.9
33		Delta 1-pyrroline-5-carboxylate synthetase B (P5CSB)	*Brassica napus*	gi|12667251	21	84	734.4054	734.3745	42.1
34		Heat shock protein (HPS)	*Medicago truncatula*	gi|355516679	12	75	802.3804	802.4912	-19.31
35		POZ/BTB-containing G-protein 1 isoform 1 (POB1)	*Theobroma cacao*	gi|508777657	1	48	1043.5313	1043.4996	30.4
36		Ribosomal protein L2, partial	*Cyrtandra* sp. *Kului 2*	gi|427338779	1	62	1406.7473	1406.8358	-10.48
37		DnaJ-like protein	*Chlamydomonas reinhardtii*	gi|158282557	1	49	1357.5482	1357.7565	-13.15
38		Heat shock cognate 70 kDa protein (HPS), putative, expressed	*Oryza sativa Japonica group*	gi|108707472	18	69	785.3783	785.3783	-63.6
39		Endo-1,4-beta-glucanase	*Gossypium hirsutum subsp. latifolium*	gi|345103975	1	47	1072.52	1072.5587	-1.11
40		POZ/BTB-containing G-protein 1 isoform 1 (POB1)	*Theobroma cacao*	gi|508777657	1	49	1043.537	1043.4996	12.32
41		Voltage-gated ion channel (VIC) superfamily	*Micromonas pusilla CCMP1545*	gi|226459371	1	42	1227.6391	1227.6935	-14.12
42		2-Oxoglutarate/malate translocator (DiT1), chloroplastic	*Triticum urartu*	gi|474119465	1	51	1043.5386	1043.6339	-10.35
43		Aux/iaa gene family member	*Oryza sativa Indica group*	gi|149391750	1	41	1466.6295	1466.7226	-23.26
44		Putative cytosolic factor	*Trifolium pratense*	gi|84453208	13	159	716.3659	716.4068	-57.14
45		Predicted protein	*Micromonas pusilla (strain CCMP1545)*	gi|226458862	28	63	704.2252	704.3341	-63.62
46		Midasin (fragment)	*Anthurium amnicola*	gi|1063020994	9	68	868.3016	868.5018	-21.42
47		Peptidyl-prolyl *cis*-*trans* isomerase	*Aralia elata*	gi|397789262	11	220	736.2067	736.3504	-21.21
48		Isoflavone 2′-hydroxylase	*Cajanus cajan*	gi|1012367600	18	74	762.2593	762.4752	-45.17
49		Uncharacterized protein	*Brachypodium distachyon*	gi|944082437	9	98	1198.4167	1198.6095	-15.43
50		Glutathione S-transferase parA-like	*Vitis vinifera*	gi|225466934	4	93	871.4382	871.4916	-11.14
51		Beta-D-xylosidase 2-like	*Cucumis sativus*	gi|449505346	2	52	1553.8539	1553.8539	-13.8
52		Calcium-binding protein CML45	*Aegilops tauschii*	gi|475525129	10	71	1044.5213	1044.4407	27.22
53		Annexin	*Manihot esculenta*	gi|1035923882	14	122	1008.4301	1008.608	-28.41
54		Linoleate 9S-lipoxygenase 5-like	*Cucumis sativus*	gi|449481072	20	72	721.3351	721.3507	-21.69
55		eIF-2-alpha kinase activator (GCN1)	*Dichanthelium oligosanthes*	gi|1070937218	31	67	749.3546	749.3919	-49.8
56		Cytochrome P450	*Cynara cardunculus var. scolymus*	gi|976915107	18	79	733.3393	733.3718	-44.39
57		Cytokinin hydroxylase-like	*Glycine max*	gi|356573678	8	77	1037.6254	1037.5618	15.21
58		Auxin-induced protein	*Aegilops tauschii*	gi|475526794	14	275	763.4072	763.3534	21.62
59		GAPC-2	*Arabidopsis lyrata subsp. lyrata*	gi|297338591	23	567	810.3852	810.4058	-25.37
60		Phospholipase D delta-like	*Solanum lycopersicum*	gi|460371003	17	77	733.4599	733.4374	30.6
61		Auxin-responsive protein	*Eucalyptus grandis*	gi|702443185	9	84	1202.4522	1202.6659	-15.34

**Table 3:** Content of protein as affected by solid-liquid ratio, microwave power, microwave time, and extraction pH in a *L*_9_(3^4^) orthogonal experiment.

Test no.	Solid-liquid ratio (*m* : *v*)	Microwave power (W)	Microwave time (s)	Extraction pH	Protein content (mg/g)
1	1 (1 : 30)	1 (210)	1 (90)	1 (10.5)	89.94
2	1	2 (280)	2 (120)	2 (11)	92.36
3	1	3 (350)	3 (150)	3 (11.5)	99.58
4	2 (1 : 35)	1	2	3	98.47
5	2	2	3	1	103.39
6	2	3	1	2	89.23
7	3 (1 : 40)	1	3	2	101.41
8	3	2	1	3	95.64
9	3	3	2	1	94.13
K1	281.88	289.82	274.81	287.46	
K2	291.09	291.39	284.96	283	
K3	291.18	282.94	304.38	293.69	
R	9.3	8.45	29.57	10.69	

## Data Availability

The data used to support the findings of this study are available from the corresponding author upon request.

## Supplementary Materials

Supplementary Materials Supplementary Table S1: volume of each reaction factor for determination of *α*-glycosidase inhibition rate. Supplementary Table S2: analytical factors and levels for the protein extraction process in a single-factor experiment. Supplementary Table S3: analytical factors and levels for the protein extraction process in a L_9_(3^4^) orthogonal experiment.
