# Supplementary material for: Seed Proteomic Profiles of Three Paeonia Varieties and Evaluation of Peony Seed Protein as a Food Product
Source: Biomed Res Int. 2020 Nov 18;2020:5271296. doi: 10.1155/2020/5271296 (PMC7695507; doi:10.1155/2020/5271296)
Supplement: Supplementary Materials — Supplementary Table S1: volume of each reaction factor for determination of α-glycosidase inhibition rate. Supplementary Table S2: analytical factors and levels for the protein extraction process in a single-factor experiment. Supplementary Table S3: analytical factors and levels for the protein extraction process in a L9(34) orthogonal experiment. [file 5271296.f1.docx]

**Supplementary Table S1 Volume of each reaction factor for determination of α-glycosidase inhibition rate.**

| **Group** | **PBS (mL)** | **PNPG (mL)** | **Sample (mL)** | **Enzyme (mL)** |
| --- | --- | --- | --- | --- |
| Control group | 1.6 | 0.2 | - | 0.2 |
| Measurement group | 1.5 | 0.2 | 0.1 | 0.2 |
| Blank group | 1.8 | 0.2 | - | - |
| Background group | 1.9 | - | 0.1 | - |

Dash represents nothing added.

**Supplementary table S2 Analytical factors and levels for protein extraction process in a single-factor experiment.**

| **Level** | **Factor** | | | |
| --- | --- | --- | --- | --- |
|  | **A solid-liquid ratio (g/ml)** | **B Microwave power (w)** | **C Microwave time (s)** | **D Extraction pH** |
| 1 | 1：25 | 210 | 30 | 9.5 |
| 2 | 1：30 | 280 | 60 | 10.0 |
| 3 | 1：35 | 350 | 90 | 10.5 |
| 4 | 1：40 | 420 | 120 | 11.0 |
| 5 | 1：45 | 490 | 150 | 11.5 |

**Supplementary Table S3 Analytical factors and levels for protein extraction process in a L_9_(3^4^) orthogonal experiment**

| **Test No.** | **A solid-liquid ratio (g/ml)** | **B Microwave power (w)** | **C Microwave time (s)** | **D Extraction pH** |
| --- | --- | --- | --- | --- |
| 1 | 1 (1：30) | 1 (210) | 1 (90) | 1 (10.5) |
| 2 | 1 | 2 (280) | 2 (120) | 2 (11) |
| 3 | 1 | 3 (350) | 3 (150) | 3 (11.5) |
| 4 | 2 (1：35) | 1 | 2 | 3 |
| 5 | 2 | 2 | 3 | 1 |
| 6 | 2 | 3 | 1 | 2 |
| 7 | 3 (1：40) | 1 | 3 | 2 |
| 8 | 3 | 2 | 1 | 3 |
| 9 | 3 | 3 | 2 | 1 |
| K1 | 281.88 | 289.82 | 274.81 | 287.46 |
| K2 | 291.09 | 291.39 | 284.96 | 283 |
| K3 | 291.18 | 282.94 | 304.38 | 293.69 |
| R | 9.3 | 8.45 | 29.57 | 10.69 |
